# Sequencing of Transcriptome and Small RNA Revealed the Xenobiotic Metabolism-Related Genes and Potential Regulatory miRNA in Asian Tramp Snail

**DOI:** 10.3389/fgene.2020.595166

**Published:** 2021-01-13

**Authors:** Qun Yang, Wanjun Yang, Feng Shang, Biyue Ding, Jinzhi Niu, Jinjun Wang

**Affiliations:** ^1^Key Laboratory of Entomology and Pest Control Engineering, College of Plant Protection, Southwest University, Chongqing, China; ^2^State Cultivation Base of Crop Stress Biology for Southern Mountainous Land, Academy of Agricultural Sciences, Southwest University, Chongqing, China

**Keywords:** *Bradybaena similaris*, cytochrome P450 monooxygenase, glutathione-S-transferase, carboxyl/cholinesterase, ATP-binding cassette transporter, RNA interference

## Abstract

The Asian tramp snail, *Bradybaena similaris* (Ferusssac), is an invasive land snail species and has been a rising agricultural pest in south of China. As a pest, it also plays a role in transmission of *Angiostrongylus cantonensis*. However, present studies on this species are rare and the molecular information is limited. For this purpose, we sequenced the transcriptome and small RNA of *B. similaris* collected from citrus orchards. In total, 89,747 unigenes with an N50 size of 1287 bp and an average length of 817 bp were generated from ∼8.9 Gb transcriptome and 31 Mb clean reads were generated from ∼36 Mb small RNA library. To demonstrate the usefulness of these two datasets, we analyzed a series of genes associated with xenobiotic metabolism and core RNAi machinery. Analysis of the transcripts resulted in annotation of 126 putative genes encoding cytochrome P450 monooxygenases (CYP, 45), carboxyl/cholinesterases (CCE, 13), glutathione-S-transferases (GST, 24), and ATP-binding cassette transporters (ABC, 44). Analysis of the small RNA detected 42 miRNAs. In addition, four genes involved in small RNA pathways (miRNA, piRNA, and siRNA) were identified, and a total of 430 genes that can be targeted by miRNAs were predicted. Moreover, we found that a few miRNAs could target certain genes involved in xenobiotic metabolism. Therefore, we believe that these two datasets and the characterization of the identified/predicted genes will facilitate the molecular study of this species as well as other land snails with agricultural importance.

## Introduction

The Asian tramp snail, *Bradybaena similaris* (Mollusca: Gastropoda: Pulmonata: Stylommatophora: Bradybaenidae), is an invasive land snail that has been a widely distributed pest. It usually inhabits garden plants, crops, and herbaceous grounds covered with organic debris. *B. similaris* is an increasingly important agricultural pest, feeding on various plants and being harmful to some economically important plants such as coffee and fruits, which has directly resulted in great economic losses (Serniotti et al., 2019). They start to come out looking for food at the beginning of March (temperature >10°C) and are most active from April to June (10–30°C) (Wang et al., 2016). In addition, this pest has also been reported as intermediate hosts of trematodes and nematodes (Alves et al., 2014; Morassutti et al., 2014), and it has been one of known intermediate hosts of *Angiostrongylus cantonensis* in China (Shan et al., 2008). Chemical molluscicides, such as niclosamide and metaldehyde, are usually applied to control snails (Lutinski et al., 2016; Wu et al., 2018). However, application of chemical agents to control pests is not sustainable due to low selectivity to target pest, resulting in pesticide resistance. Moreover, it can contaminate the environment and affect the beneficial animals (Lutinski et al., 2016; Tunholi et al., 2017). Recently, studies were reported showing that plant-derived molluscicides have obviously molluscicidal activity in the laboratory (Jia et al., 2019; Ke et al., 2019). It should be highlighted that the compounds present in the composition of plant-derived molluscicides are complex and some of them have high toxicity to non-target organisms, so evaluation and demonstration of the eco-toxicity information will be needed before using in the field (Pereira et al., 2020).

To better understand the molluscicidal activity of chemical pesticides or plant secondary metabolism, it is necessary to understand the molecular information of xenobiotic metabolism on snail. The molecular mechanism of xenobiotic detoxification can be divided into three phases (Van Leeuwen and Dermauw, 2016). In phase I, cytochrome P450 monooxygenases (CYPs) and carboxyl/cholinesterases (CCEs) can alter the structures of toxic compounds by introducing a nucleophilic functional group to produce a compound with higher activity and water solubility. Then, in phase II, the enzymes such as glutathione-S-transferases (GSTs) reinforce the water solubility by conjugation with endogenous molecules. In phase III, cellular transporters, such as ATP-binding cassette transporters (ABCs), transport all conjugates out of the cell. These genes involved in xenobiotic detoxification also showed a link between host plant adaptation and pesticides resistance (Dermauw et al., 2013). However, the molecular information of *B. similaris* is rare, which has hampered the identification of the genes involved in xenobiotic detoxification. In addition, these genes are also potential targets of RNA interference (RNAi) in enhancing toxicity of pesticides and host plant secondary metabolites (Niu et al., 2018).

RNA interference, based on post-transcriptional gene silencing, is a promising novel approach for controlling pest and a sustainable way in plant protection (Liu et al., 2020; Yan et al., 2020). However, the machinery of RNAi pathway in *B. similaris* is still unknown. RNAi is classified into three pathways, including small-interfering RNA (siRNA), microRNA (miRNA), and piwi-interacting RNA (piRNA). Briefly, for siRNA pathway, Dicer 2 cleavages the long dsRNA into 19- to 23-nt double-stranded siRNAs. These siRNAs then load to Argonaute 2 centered RNA-induced silencing complex (RISC), which finally inhibits the expression of the target gene. For miRNA pathway, primary RNAs are processed by Drosha and Pasha into shorter precursor miRNAs, then followed by the slicing of Dicer 1 into (21–24 nt) mature miRNAs. For piRNA pathway, the precursor piRNAs are cut into 26- to 32-nt piRNAs by Argonaute 3 to generate antisense piRNAs. These antisense piRNAs are trimmed by Aubergine and P-element induced wimpy testis (Piwi) and then generate sense piRNAs to create a feedback loop, known as ping-pong cycle (Dowling et al., 2016; Cooper et al., 2019).

In this study, we are aiming to provide the datasets of deep sequenced transcriptome and small RNA. The usefulness of these datasets will be demonstrated to identify genes involved in xenobiotic detoxification as well as the potential regulatory miRNAs. We believe that this study will facilitate the molecular study of this species as well as other land snails with agricultural importance.

## Materials and Methods

### Sample Collection

**FIGURE 1:**
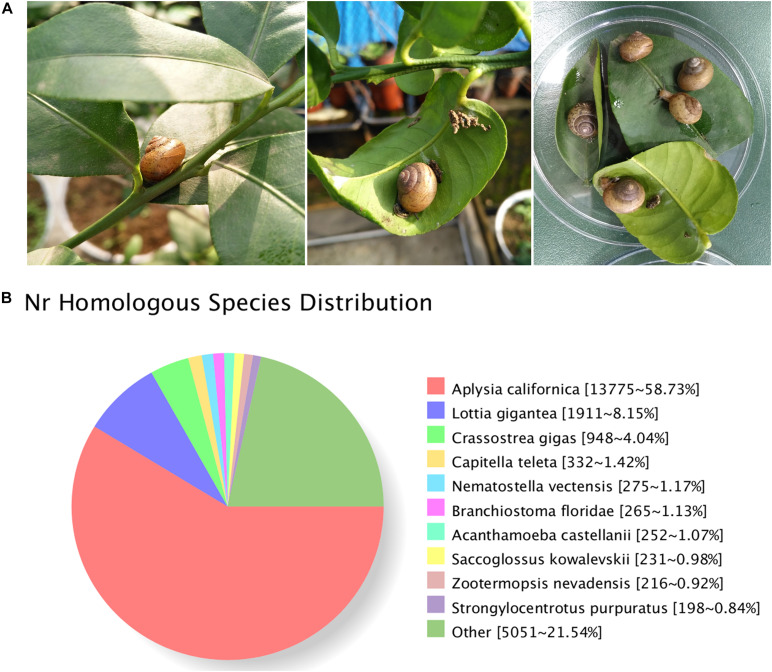
**(A)** *Bradybaena similaris* and citrus leaves. **(B)** Homology analysis of unigenes for *B*. *similaris*.

The Asian tramp snail *B. similaris* (Figure 1A) were collected from the citrus orchards in the Citrus Research Institute of Chinese Academy of Agricultural Sciences (CAAS), located on the Beibei District, Chongqing, China, in early spring (March) 2017. To avoid potential contamination from host plants, only the foot muscle from 10 adult snails were dissected for RNA extraction.

### Transcriptome and Small RNA Sequencing

After washing with chilled PBS (pH 7.4) three times, the dissected foot muscles were homogenized in TRIzol reagent (Invitrogen, Carlsbad, CA, United States) to isolate RNA under the manufacturer’s protocol. About 1% agarose gel and a Nanodrop ONE spectrophotometer (GE Healthcare Bio-Science, Uppsala, Sweden) were used to confirm RNA quality and quantity, respectively. The library was constructed using a TruSeq Total RNA Sample Prep Kit (Illumina, San Diego, CA, United States). Then, sequencing was performed with PE150 in a HiSeq X Ten platform (Illumina). Low-quality reads and reads with adaptors and poly-N were removed from the raw reads. Then, these high-quality reads were analyzed with the Trinity program.^1^ The obtained sequence processed by Trinity, which could not be extended on either end, was called unigene. After that, those assembled unigenes were annotated with a cutoff *E*-value of 10^–5^ using BLASTx search against the following databases: NR; GO; COG/KOG/eggNOG; KEGG; Pfam; Swiss-Prot.

The small RNA library for *B. similaris* was constructed with small RNAs (18–30 nt) by PE50 in a HiSeq X Ten platform. Reads with adaptors, poly-N, low quality were removed. Then, clean reads were mapped to *B. similaris* transcriptome database using Bowtie software (version 1.0). Sequence alignment of the clean reads was carried out with the following database: Silva,^2^ GtRNAdb,^3^ Rfam,^4^ and Repbase^5^ to filter ribosomal RNA (rRNA), transfer RNA (tRNA), small nuclear RNA (snRNA), small nucleolar RNA (snoRNA), and other ncRNA and repeats. Subsequently, the remaining clean reads were annotated by miRBase (V21) to detect known miRNAs. Novel miRNA secondary structures were predicted by Ranfold tools software (Version 2.0). MiRanda (Version 3.3) and RNAhybrid (Version 2.1) were used to predict miRNA targets.

### Identification of Xenobiotic Metabolism Related Genes and Core RNAi Machinery

We retrieved transcripts encoding putative *B. similaris* genes related to xenobiotic metabolism (including CYPs, CCEs, GSTs, and ABCs) and core RNAi machinery (including miRNA, siRNA, and piRNA pathway) from *B. similaris* transcriptome. Firstly, BlastStation-Local 64 software was used to screen candidate unigenes associated with xenobiotic metabolism and candidate unigenes associated with core RNAi machinery. Known sequences from arthropods including *Drosophila melanogaster*, *Tribolium castaneum*, *Apis mellifera*, *Bombyx mori*, and *Tetranychus urticae*, and molluscs including *Aplysia californica* and *Biomphalaria glabrata* were used as queries for the homology searches. The matching sequences were then translated using the ExPASy-Translate tool^6^ and manually checked. The complete coding region was determined according to the open reading frame (ORF) finder.^6^ Finally, the selected candidate sequences of *B. similaris* with a minimum ORF length (CYPs: 200 nt; CCEs: 300 nt; GSTs: 200 nt) were aligned with known genes from other arthropods and molluscs using Mega 7.0 with the default settings in ClustalW for phylogenetic analysis (Kumar et al., 2016). For *B. similaris* ABCs, we used the nucleotide-binding domain (NBD) for phylogenetic analysis and extracted the NBDs using NCBI BLAST.^7^ A Neighbor-Joining analysis was performed using bootstrapping with 1000 replicates to construct the phylogenetic trees.

## Results

### Overview of Transcriptome

A total of 29,809,392 clean reads were generated (NCBI accession number SRR6981555) for *B. similaris*, encompassing about 8.91 Gb sequencing clean data with a GC content (guanine–cytosine content) of 44.97%. More than 89% clean reads showed quality scores higher than the Q30 (base calling accuracy of 99.9%). These clean reads were further assembled into 89,747 unigenes with an average length of 817 bp and an N50 size (at which 50% of assembly was covered) of 1287 bp (Table 1 and Supplementary Figure 1). Among 89,747 unigenes, a total of 26,331 unigenes were annotated against various databases (including NR, GO, COG, KOG, eggNOG, KEGG, Pfam, and Swiss-Prot) (Supplementary Table 1). Out of 89,747 unigenes, 23,481 unigenes (26.16%) were matched against the NCBI non-redundant (NR) protein database (Table 1 and Supplementary Table 1). According to the best hit in the NR protein database, 13,775 unigenes (58.73% of total NR annotation unigenes) had a strong homology with the California sea hare *A. californica*, followed by the owl limpet *Lottia gigantea* (1911 unigenes, 8.15%), the oyster *Crassostrea gigas* (948 unigenes, 4.04%), deposit feeder *Capitella teleta* (332 unigenes, 1.42%), the sea anemone *Nematostella vectensis* (275 unigenes, 1.17%), etc. (Figure 1B).

**TABLE 1:** Summary of the transcriptome and small RNA libraries from *Bradybaena similaris*.

	**Type**	**Counts**	**Percentage**
Transcriptome	Total number of clean reads	29,809,392	100
	Total clean nucleotides (bp)	8,912,995,154	100
	Q30 percentage	/	89.66
	GC percentage	/	44.97
	Number of unigenes	89,747	/
	Total length of unigenes (bp)	73,297,693	/
	N50 length of unigenes (bp)	1287	/
	Mean length of unigenes (bp)	817	/
	Unigenes annotations against NR	23,481	26.16
	Unigenes annotations against Swiss-Prot	12,463	13.89
	Unigenes annotation against KEGG	10,908	12.15
	Unigenes annotations against GO	8240	9.18
	Unigenes annotations against COG	7744	8.63
Small RNA tags	Total reads	36,340,042	100
	Clean reads	31,456,038	86.56
	Q30 percentage	/	98.56
	Mapped to transcriptome	6,443,477	17.73

Based on the analysis of GO annotation, a total of 8240 unigenes were classified into cellular component, molecular function, and biological process (Supplementary Figure 2). The largest category was biological process (including 18 subcategories; 20,222 sequences, 48.48%), followed by the cellular component (including 18 subcategories; 11,644 sequences, 27.92%) and molecular function (including 16 subcategories; 9845 sequences, 23.60%) (Supplementary Table 2). A total of 7744 unigenes were grouped into 25 COG categories (Supplementary Figure 3). The “general function prediction” was the most common category (1563, 20.18%), followed by “translation, ribosomal structure and biogenesis” (791, 10.21%), and “posttranslational modification, protein turnover, chaperones” (674, 8.70%). A total of 10,908 *B. similaris* unigenes were mapped onto 273 KEGG pathways. The most abundant pathway was “ribosome” (469, 4.30%) from Genetic Information Processing, followed by “protein processing in endoplasmic reticulum” (289, 2.26%) from Genetic Information Processing and “purine metabolism” (247, 2.26%) from Metabolism (Supplementary Figure 4).

### Identification of Genes Potentially Involved in Xenobiotic Metabolism and Core RNAi Machinery

**FIGURE 2:**
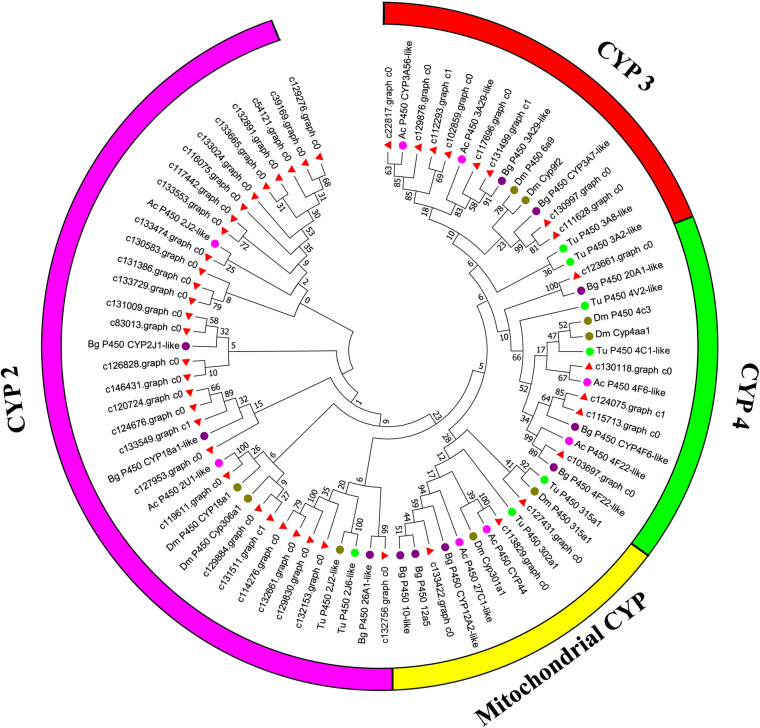
Phylogenetic analysis of putative cytochrome P450 monooxygenase (CYP) genes. Neighbor-joining tree of *B. similaris, Aplysia californica* (Ac), *Biomphalaria glabrata* (Bg), *Drosophila melanogaster* (Dm), and *Tetranychus urticae* (Tu) CYP protein sequences. Numbers at each branch indicate the percentage of the times a node was supported in 1000 bootstrap pseudo-replications by the neighbor-joining method. The sequences used for phylogenetic tree construction are listed in Supplementary Table 3.

**FIGURE 3:**
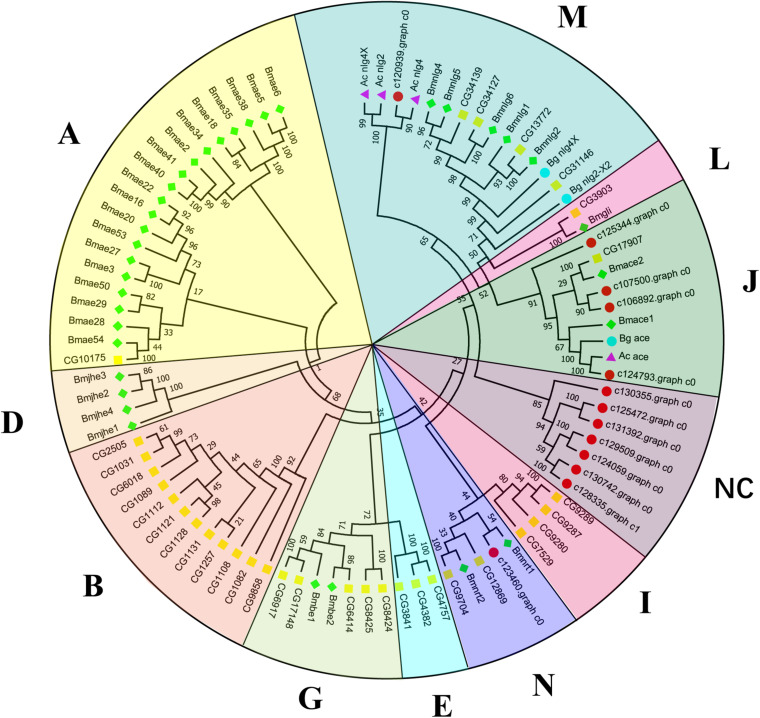
Phylogenetic analysis of putative carboxyl/cholinesterase (CCE) genes. Neighbor-joining tree of *B. similaris, Aplysia californica* (Ac), *Biomphalaria glabrata* (Bg), *Drosophila melanogaster* (Dm), and *Bombyx mori* (Bm) CCE protein sequences. Numbers at each branch indicate the percentage of the times a node was supported in 1000 bootstrap pseudo-replications by the neighbor-joining method. The sequences used for phylogenetic tree construction are listed in Supplementary Table 4.

**FIGURE 4:**
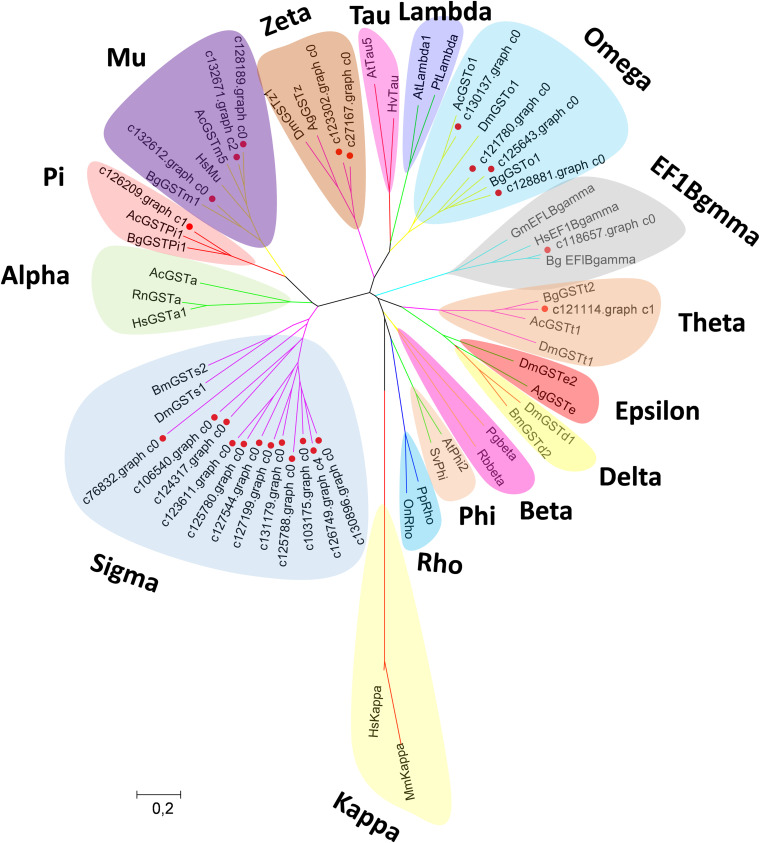
Phylogenetic analysis of *B*. *similaris* glutathione S-transferases (GSTs). The phylogenetic tree was supported in 1000 bootstrap pseudo-replications by the neighbor-joining method. GenBank accession numbers for the sequences used in the phylogenetic tree are as follows: *A. californica* (Ac) Theta, XP_005089374; *B. glabrata* (Bg) Theta, XP_013081705; *D. melanogaster* (Dm)Theta, NP_610509; *A. californica* Mu, XP_005093728; *B. glabrata* Mu, XP_013097111; *Thais clavigera* Mu, ACD13785; *T. urticae* (Tu) Mu, XP_015781114; *A. californica* Pi, XP_005099402; *B. glabrata* Pi, XP_013095077; *Homo sapiens* (Hs) Omega, NP_004823; *B. glabrata* Omega, XP_013062909; *D. melanogaster* Omega, NP_648237; *D. melanogaster* Sigma, NP_523767; *Bombyx mori* Sigma, NP_001036994; *D. melanogaster* Delta, NP_524326; *B. mori* Delta, NP_001036974; *D. melanogaster* Epsilon, NP_611323; *Anopheles gambiae* (Ag) Epsilon, XP_319969; *H. sapiens* Alpha, NM145740; *Rattus norvegicus* Alpha, AAF37739; *A. californica* Alpha, XP_012945278; *D. melanogaster* Zeta, NP_649894; *A. gambiae* Zeta, XP_312009; *Photobacterium ganghwense* Beta, PSU09724; *Rhizobiales bacterium* Beta, WP_113235669; *Arabidopsis thaliana* Phi, NP_192161; *Silene vulgaris* Phi, AAA33931; *A. thaliana* Tau, NP_180506; *Hordeum vulgare* Tau, BAE44477; *A. thaliana* Lambda, NM_001125685; *Pinus tabuliformis*, Lambda, AGC13140; *Pimephales promelas* Rho, AAF78081; *Oreochromis niloticus* Rho, ABV24480; *Glycine max* EF1Bgamma, NP_001241223; *B. glabrata* EF1Bgamma, XP_013072347; *H. sapiens* EF1Bgamma, NP_001395; *Mus musculus* Kappa; and *H. sapiens* Kappa, AAS01180. The sequences used for phylogenetic tree construction are listed in Supplementary Table 5.

**FIGURE 5:**
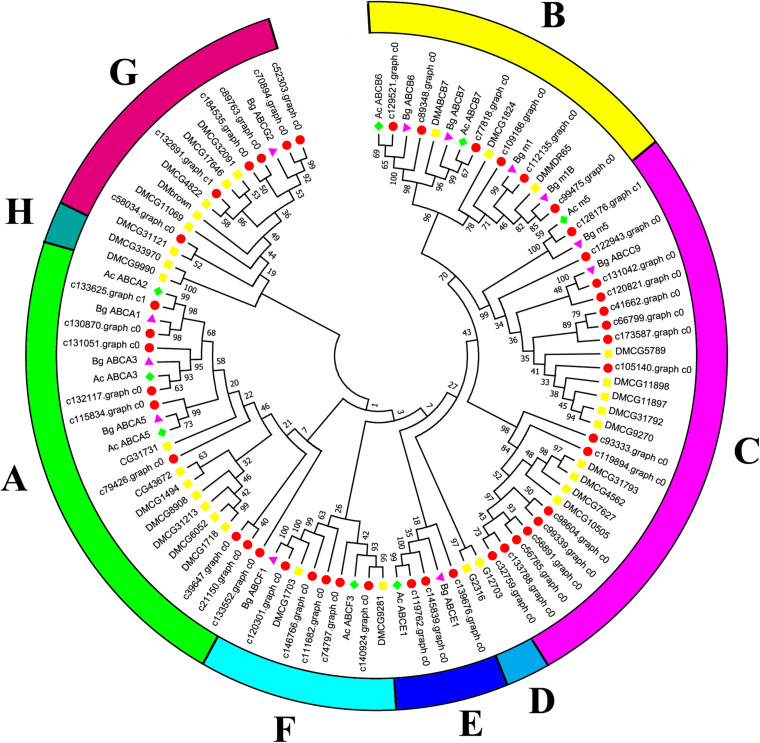
Phylogenetic analysis of *B. similaris* ABC protein NBDs. A set of ABC protein NBDs extracted from *B. similaris*, *D. melanogaster* (Dm), *A. californica* (Ac), and *B. glabrata* (Bg) were used into phylogenetic analysis. Numbers at each branch indicate the percentage of the times a node was supported in 1000 bootstrap pseudo-replications by the neighbor-joining method. The sequences used for phylogenetic tree construction are listed in Supplementary Table 6.

Canonical xenobiotic metabolism genes including CYPs, CCEs, GSTs, and ABCs were analyzed based on the constructed transcriptome. In total, we identified 45 putative P450 genes. CYP2 clade has the majority P450 genes (29 genes), followed by CYP3 clade (eight genes), CYP4 clade (five genes), and Mitochondrial (three genes) (Figure 2 and Supplementary Table 3). A total of 13 putative CCEs genes were identified and grouped into four clades: J clade (four putative genes), N clade (one putative gene), M clade (one putative gene), and NC clade (seven putative genes) (Figure 3 and Supplementary Table 4). To better characterize GSTs in *B. similaris*, known genes from humans, plants, bacteria, and other animal species were also included to construct the phylogenetic tree. In total, 24 GST genes were assigned to seven classes: sigma (12 genes), pi (one gene), mu (three genes), zeta (two genes), omega (four genes), elongation factor 1-gamma (EF1Bgamma) (one gene), and theta (one gene) (Figure 4 and Supplementary Table 5). A total of 44 ABCs of *B. similaris* were assigned to subfamily A (nine genes), subfamily B (six genes), subfamily C (16 genes), subfamily E (three genes), subfamily F (five genes), and subfamily G (five genes) (Figure 5 and Supplementary Table 6).

By using the homology genes in insects including *T. castaneum*, *D. melanogaster*, *B. mori*, and *A. mellifera*, we identified four core RNAi machinery, including two Aubergine from piRNA pathway, one Argonaute 1 from miRNA pathway, and one Dicer 2 from siRNA pathway from *B. similaris* (Supplementary Figure 5 and Table 7).

### MiRNA Annotation and Potential Targets Prediction

**FIGURE 6:**
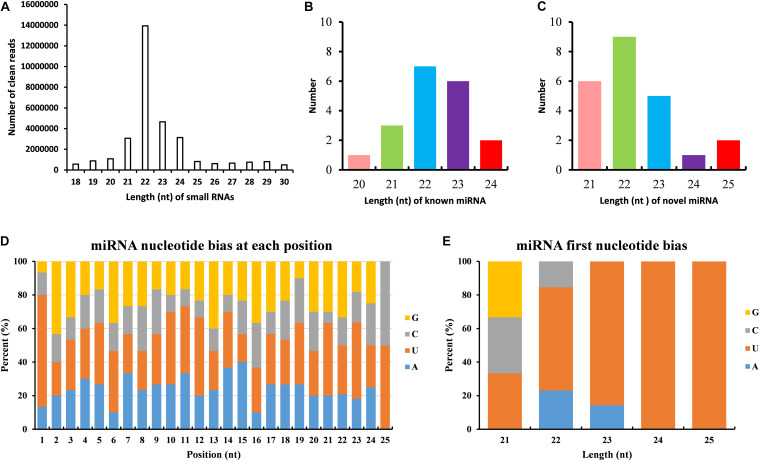
Analysis of miRNAs length and base bias of *B. similaris*. **(A)** Length of all miRNAs. **(B)** Length of known miRNAs. **(C)** Length of novel miRNAs. **(D)** Analysis of the nucleotide bias at each position of the miRNAs. **(E)** Analysis of the nucleotide bias at the first position of the miRNAs.

The dataset generated 31,456,038 (86.56%) clean reads from 36,343,042 raw reads (NCBI accession number SRR6981556), of which 6,443,477 (17.73%) reads matched to reference transcriptome (Table 1). The sequence length of most small RNA was between 21 and 24 nt, of which sequence length in 22 nt was abundant (Figure 6A). MiRNAs were annotated based on the miRBase (version 21.0) database^8^; if the sequence completely matched a known miRNA, we then assigned the known miRNA name to the candidate *B. similaris* miRNA. If the sequence matched the seed regions (bases 2–8) but did not exactly match the remaining bases of the known miRNA, we then regarded it as novel miRNA. In total, we obtained 42 miRNA in *B. similaris*, including 19 known miRNAs (Table 2 and Figure 6B) and 23 novel miRNA (Supplementary Table 8 and Figure 6C). The preferred bases of *B. similaris* miRNA at the seed region positions that play an important role in miRNA complementary and target recognition were G(guanine), G, A(adenine)/U(uracil), U, U/G, A, and C(cytosine)/G (Figure 6D). The first base of the miRNAs (21–25 nt) indicated that U was the most used base while G was the least used base. The most common first base of the miRNAs with 22 nt was U (61.54%) (Figure 6E), which is the most preferred nucleotide for Dicer while recognizing and trimming the precursor miRNA.

**TABLE 2:** List of known miRNAs from *Bradybaena similaris*.

**miRNAs**	**Mature arm**	**Reads**	**Sequences**	**Length (bp)**	**Best match species**
*bsi-miR-1-1*	3p	1,696,594	uggaauguaaagaaguauguau	22	*Lottia gigantea*
*bsi-miR-10a*	5p	297,002	uacccuguagauauccgaauuugu	24	*Melibe leonina*
*bsi-miR-1-2*	3p	98,270	uggaauguaaagaaguaugu	20	*Caenorhabditis elegans*
*bsi-miR-1-3*	3p	98,270	uggaauguaaagaaguaugua	21	*Caenorhabditis elegans*
*bsi-miR-8-1*	3p	47,644	uaauacugucagguaaagaugu	22	*Drosophila melanogaster*
*bsi-miR-8-2*	3p	47,644	uaauacugucagguaaagauguc	23	*Drosophila melanogaster*
*bsi-miR-2d*	3p	2413	uaucacagccugcuugguucagu	23	*Lottia gigantea*
*bsi-miR-219*	3p	1675	agaacuguguguggacaucagu	22	*Melibe leonina*
*bsi-miR-2b*	3p	1299	ucacagccagcuuugaugagc	21	*Manduca sexta*
*bsi-miR-2a-1*	3p	1285	uaucacagccagcuuugaugagcg	24	*Acyrthosiphon pisum*
*bsi-miR-7*	5p	996	uggaagacuagugauuuaguugu	23	*Melibe leonina*
*bsi-miR-1*	5p	969	acauacuucuuugcuaucccau	22	*Melibe leonina*
*bsi-miR-2a-2*	3p	854	uaucacagccagcuuugaugagc	23	*Drosophila melanogaster*
*bsi-miR-8*	5p	670	cgucuuaccuagcagcauugga	22	*Melibe leonina*
*bsi-miR-2c*	3p	148	uaucacagccagcuuugaugggc	23	*Drosophila melanogaster*
*bsi-miR-219-1*	5p	33	ugauuguccaaacgcaauucuug	23	*Aedes aegypti*
*bsi-miR-219-2*	5p	32	ugauuguccaaacgcaauucu	21	*Drosophila melanogaster*
*bsi-miR-219-3*	5p	32	ugauuguccaaacgcaauucuu	22	*Drosophila pseudoobscura*
*bsi-miR-1*	5p	9	ugggauagcaaagaaguauguu	22	*Melibe leonina*

To validate the possible biological context of the identified *B. similaris* miRNA for future study, we predicted the miRNA target from the small RNA dataset. A total of 91 genes and 341 genes could be targeted by 15 conserved known miRNA and 19 novel miRNA, respectively (Supplementary Tables 9, 10). Interestingly, certain gene xenobiotic metabolism can be targeted by a few novel miRNAs (Supplementary Table 11). One CYP gene (c131511.graph_c1) from CYP2 clade can be targeted by *bsi-miR-novel 5*, two ABCs (c122943.graph_c0, c98604.graph_c0) from ABC subfamily C can be targeted by *bsi-miR-novel 6*, and one CCE (c106892.graph_c0) from clade J can be targeted by *bsi-miR-novel 8*. This indicated that the miRNAs in *B. similaris* are potentially involved in the regulation of xenobiotic metabolism.

## Discussion

Due to problems caused by intensive application of chemical pesticides, it is crucial to develop novel and effective alternative pest management strategies. RNAi is one of the most promising novel approaches for pest control, which triggers toxic effects in the target species without harming non-target organisms in the ecosystem (Christiaens et al., 2018). However, lack of genetic background for some pest, including the rising agricultural pest *B. similaris* in current research, has hampered in-depth studies and the development of better control strategies. Therefore, we investigated and described the results of a *de novo* deep sequencing of *B. similaris* transcriptome (8.91 Gb) and small RNAs (36.34 M) with Illumina sequencing technology.

In the transcriptome, a total of 89,747 unigenes were generated. The number of unigenes in our study is greater than those assembled in the marine gastropod *Hemifusus tuba* (61,575) (Li et al., 2019) but smaller than the snail *Satsuma myomphala* (103,744) (Kang et al., 2017), *Echinolittorina malaccana* (115,211) (Wang et al., 2014), and the rock scallop *Crassadoma gigantea* (96,006) (Cao et al., 2018). Mollusca is the second most species-rich phylum after Arthropoda in the animal kingdom, yet only a few genomic information has been published so far. For gastropods, the already sequenced genomes could be found in the sea hare *A. californica* (Moroz et al., 2006); the freshwater snail *B. glabrata* (Adema et al., 2017), *Radix auricularia* (Schell et al., 2017), and *Lymnaea stagnalis* (unpublished; genome data are available in the NCBI database: GCA_900036025.1); the sea snail *Patella vulgata* (Kenny et al., 2015), *Conus tribblei* (Barghi et al., 2016), and *L. gigantea* (Simakov et al., 2013); and the shelled pteropod *Limacina bulimoides* (Choo et al., 2020). In this study, out of 23,481 annotated unigenes against the NR protein database, 13,775 unigenes (58%) have strong homology with *A. californica*. Because these two species are distantly related organisms, the proportion is normal when compared with the sequenced transcriptome in other molluscs. The land snail *S. myomphala* showed 51% homologous unigenes with *A. californica* (Kang et al., 2017) and the freshwater mussel *Cristaria plicata* showed 40% homologous unigenes with the oyster *C. gigas* (Patnaik et al., 2016).

Xenobiotic metabolism genes on arthropods (i.e., insects and mites) have been well documented. However, they received little attention on molluscs and the majority of studies focused on the role in environmental pollutant metabolism, especially polycyclic aromatic hydrocarbons (Rewitz et al., 2006). For example, the snail *Xeropicta derbentina* was recommended as biomarkers monitoring pesticide exposure in the field because their B-type esterases are sensitive to organophosphorus insecticides (Laguerre et al., 2009). Based on the phylogenetic analysis, which is helpful for investigating the evolutionary relationships between genes among species, we identified a total of 126 genes related to potential xenobiotic metabolism, including 45 P450s, 13 CCEs, 24 GSTs, and 44 ABCs. According to the phylogenetic analysis, the total number of P450 unigenes in clade CYP2 (29 unigenes, 64%) is greater than other clades, which indicates that P450s in *B. similaris* were expanded in clade CYP2 when compared with *B. glabrata* (99 P450s, of which 33 in clade CYP2 and 35 in clade CYP3) (Adema et al., 2017) and arthropods, which have the majority of P450s in clade CYP3 and clade CYP4 such as mites (Bajda et al., 2015) and insects (Ai et al., 2011; Yu et al., 2015). Although most of them in *B. similaris* showed high homology with 2J2-like genes from *A. californica* and *T. urticae*, further studies are still needed to confirm this. Half of GSTs belong to Sigma subfamily, which may indicate that these genes in this subfamily are major detoxification genes. The clarification and characterization of these genes involved in the xenobiotic mechanism will be helpful to future studies in understanding the response of target genes to pesticides.

As a powerful gene function study tool, RNAi is widely used in insects that are also intensively explored to develop novel pest control strategy (Niu et al., 2018). Currently, RNAi are intensively investigated in invertebrates but relatively rare in molluscs (only 14 species reported) (Owens and Malham, 2015). Despite the limited studies, the successful case of RNAi-mediated gene silencing (by soaking) in the freshwater snail, *B. glabrata*, proved that RNAi is an achievable method for controlling snail pests (Knight et al., 2011). By using model insects as reference, we identified four genes associated with the core machinery RNAi pathways from the transcriptome of *B. similaris*, including one in miRNA pathway, one in siRNA pathway, and two in piRNA pathway. There were nine genes in *B. glabrata*, of which five were in miRNA pathway and four in piRNA pathway but no gene was reported in siRNA pathway (Adema et al., 2017). Although the full core RNAi machinery was not yet identified in *B. similaris*, it seemed that three RNAi pathways were conserved in this snail and provided confidence for further experimental assay to test the efficiency of RNAi in this species.

miRNA can regulate the post-transcription by repressing protein synthesis by either inhibiting the translation of mRNA or increasing mRNA degradation (O’Brien et al., 2018). We identified 42 miRNAs based on the constructed small RNA library, which is less than the number of miRNA in *P. vulgata* (45), *L. gigantea* (59), and *B. glabrata* (209) (Kenny et al., 2015; Adema et al., 2017). We found that a few xenobiotic mechanism genes can be targeted by miRNAs, which implied that miRNAs in *B. similaris* can regulate xenobiotic metabolism genes. Further experimental validations are required to confirm their involvement in pesticide detoxification.

## Conclusion

In this study, we sequenced the transcriptome and small RNA of a rising agricultural pest *B. similaris*. These two datasets were useful in identifying molecular information for certain interesting genes, which were further demonstrated for the identification of the genes potentially involved in the xenobiotic metabolism and core RNAi machinery. In addition, miRNAs of this snail were also investigated, and the prediction of miRNAs in the regulation of xenobiotic metabolism genes was analyzed. However, these two datasets only serve as the starting point of molecular research in *B. similaris*; further characterization of xenobiotic metabolism genes is still required by experimental approach, such as RNAi-based gene function study. We believe that these two datasets could not only facilitate further research of the molecular understanding of *B. similaris* but also be used as a resource for the development of novel snail pest control approach.

## Data Availability Statement

The datasets generated for this study can be found in the SRA (SRR6981555 and SRR6981556).

## Author Contributions

QY and JN conducted the design. QY and BD contributed to the experiments. QY, WY, and FS conducted the statistical analysis. QY drafted the manuscript. JN and JW contributed to proofreading and editing this written work. All authors approved the final draft of the manuscript.

## Conflict of Interest

The authors declare that the research was conducted in the absence of any commercial or financial relationships that could be construed as a potential conflict of interest.

## Footnotes

1 http://github.com/trinityrnaseq/trinitynaseq/wiki

2 http://www.arb-silva.de/

3 http://lowelab.ucsc.edu/GtRNAdb/

4 http://rfam.xfam.org/

5 http://www.girinst.org/repbase/

6 https://web.expasy.org/translate/

7 https://blast.ncbi.nlm.nih.gov/Blast.cgi

8 http://www.mirbase.org/
