# Supplementary material for: Sequencing of Transcriptome and Small RNA Revealed the Xenobiotic Metabolism-Related Genes and Potential Regulatory miRNA in Asian Tramp Snail
Source: Front Genet. 2021 Jan 13;11:595166. doi: 10.3389/fgene.2020.595166 (PMC7838618; doi:10.3389/fgene.2020.595166)
Supplement: Supplementary file 1 [file Table_1.pdf]

Supplementary material

Supplementary Tables

Table 1 Summary information of the annotated unigenes of *B. similaris* against various databases.

Annotation_Database	Annotated_Number	300 ≤ Length ≤ 1000	Length ≥ 1000
NR	23,841	8172	13,530
COG	7744	2514	4731
GO	8240	2850	4786
KEGG	10,908	3469	6834
KOG	15,381	4877	9418
PFAM	18,345	5836	11,468
Swissprot	12,463	3683	8145
eggNOG	21,704	7558	12,203
All_Annotated	26,331	9759	13,958

Table 2 Subcategories of GO annotation for *B. similaris.*

GO classify1	Number of GO category	GO classify2	*Bradybaena similaris* Unigene	
**Cellular component**	18	extracellular region	83	0.71%
		collagen trimer	4	0.03%
		cell	2638	22.66%
		membrane	1107	9.51%
		virion	3	0.03%
		cell junction	72	0.62%
		extracellular matrix	19	0.16%
		membrane-enclosed lumen	144	1.24%
		macromolecular complex	1342	11.53%
		organelle	1858	15.96%
		extracellular matrix part	8	0.07%
		extracellular region part	39	0.33%
		organelle part	874	7.51%
		virion part	3	0.03%
		membrane part	634	5.44 %
		synapse part	81	0.70%
		cell part	2635	22.63%
		synapse	100	0.86%
**Subtotal**			**11,644**	**27.92%**
**Molecular function**	16	protein binding transcription factor activity	25	0.25%
		nucleic acid binding transcription factor activity	167	1.69%
		catalytic activity	4339	44.07%
		receptor activity	143	1.45%
		guanyl-nucleotide exchange factor activity	17	0.17%
		structural molecule activity	452	4.59%
		transporter activity	454	4.61%
		binding	3846	39.07%
		electron carrier activity	58	0.58%

GO classify1	Number of GO category	GO classify2	*Bradybaena similaris* Unigene	
		antioxidant activity	42	0.43%
		channel regulator activity	2	0.02%
		metallochaperone activity	3	0.03%
		enzyme regulator activity	76	0.77%
		protein tag	1	0.01%
		translation regulator activity	2	0.02%
		molecular transducer activity	218	2.21%
**Subtotal**			**9845**	**23.60%**
**Biological process**	18	reproduction	238	1.18%
		immune system process	39	0.19%
		metabolic process	5343	26.42%
		cellular process	4220	20.87%
		reproductive process	183	0.90%
		biological adhesion	41	0.20%
		signaling	679	3.36%
		multicellular organismal process	733	3.62%
		developmental process	714	3.53%
		growth	89	0.44%
		locomotion	218	1.08%
		single-organism process	3320	16.42%
		rhythmic process	16	0.08%
		response to stimulus	972	4.81%
		localization	1116	5.52%
		multi-organism process	186	0.92%
		biological regulation	1414	6.99%
		cellular component organization or biogenesis	701	3.47%
**Subtotal**			**20,222**	**48.48%**
**Total**	**52**		**41,711**	

Table 3 Annotation details of cytochrome P450 monooxygenase (CYP) sequences used to construct a phylogenetic tree (XLSX).

Table 4 Annotation details of carboxyl/cholinesterase (CCE) sequences used to construct a phylogenetic tree (XLSX).

Table 5 Annotation details of glutathione-S-transferase (GST) sequences used to construct a phylogenetic tree (XLSX).

Table 6 Annotation details of ATP-binding cassette transporter (ABC) sequences used to construct a phylogenetic tree (XLSX).

Table 7 Annotation details of sequences of the genes associated with core machinery RNAi pathway used for phylogenetic tree construction (XLSX).

Table 8 List of novel miRNAs from *B. similaris* (XLSX).

Table 9 Target gene number prediction statistics of miRNA from *B. similaris*.

Types	All miRNA	miRNA with target	Target gene*
Known miRNA	19	15	91 (109)
Novel miRNA	23	19	341 (344)
Total	42	34	430 (453)

* The number within brackets represent the total number of *B. similaris* miRNA targets, while the number without brackets represent the number of *B. similaris* miRNA targets after removing the duplicate target genes, which indicated that the one target can be regulated by different miRNA.

Table 10 Summary information of miRNA target prediction (XLSX).

Table 11 Summary of miRNAs that could target xenobiotic metabolism genes (XLSX).

Supplementary Figures

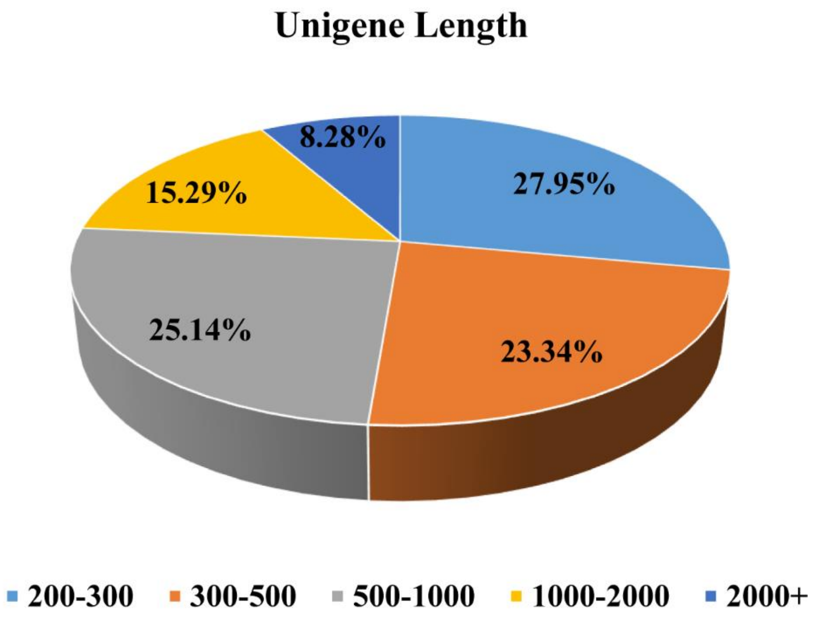

Figure 1 Unigenes length of *B. similaris*.

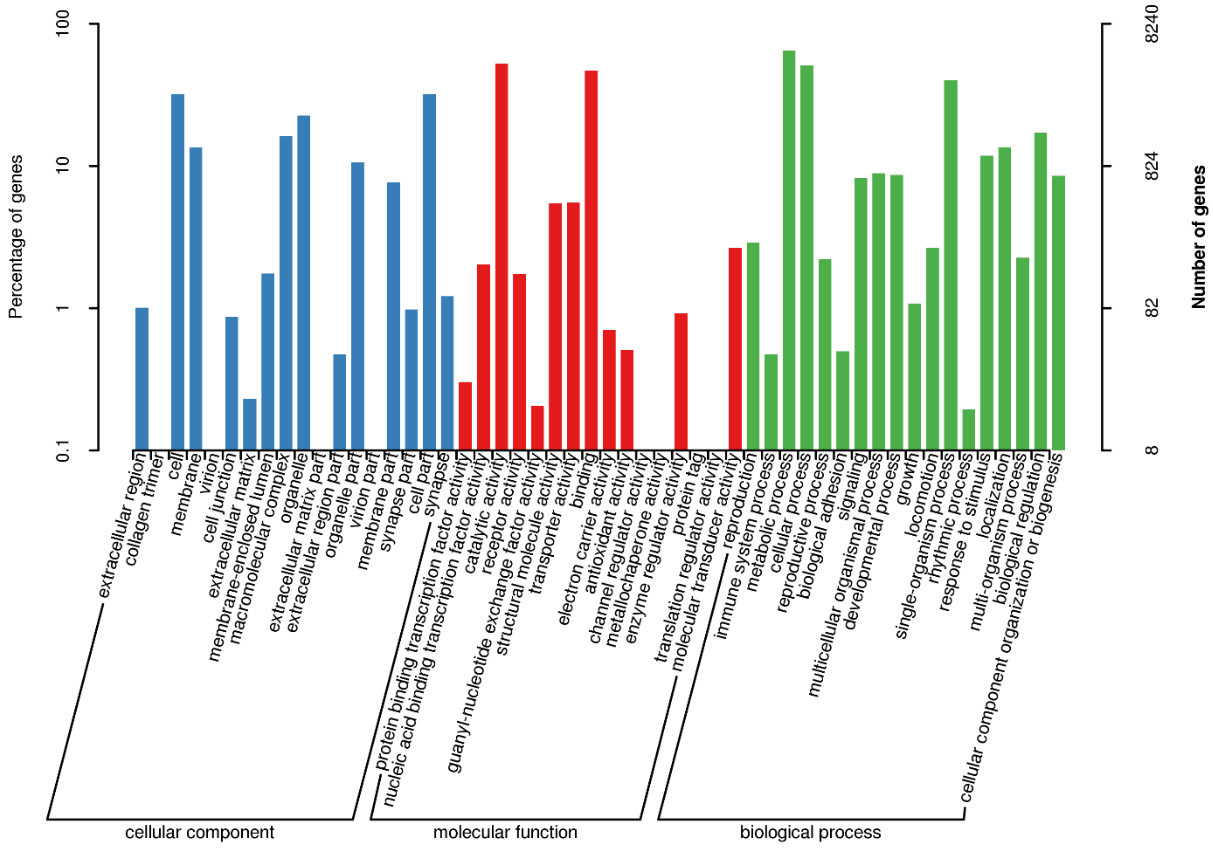

Figure 2 Gene Ontology (GO) terms for the transcriptomic sequences of *B. similaris*.

frontiers

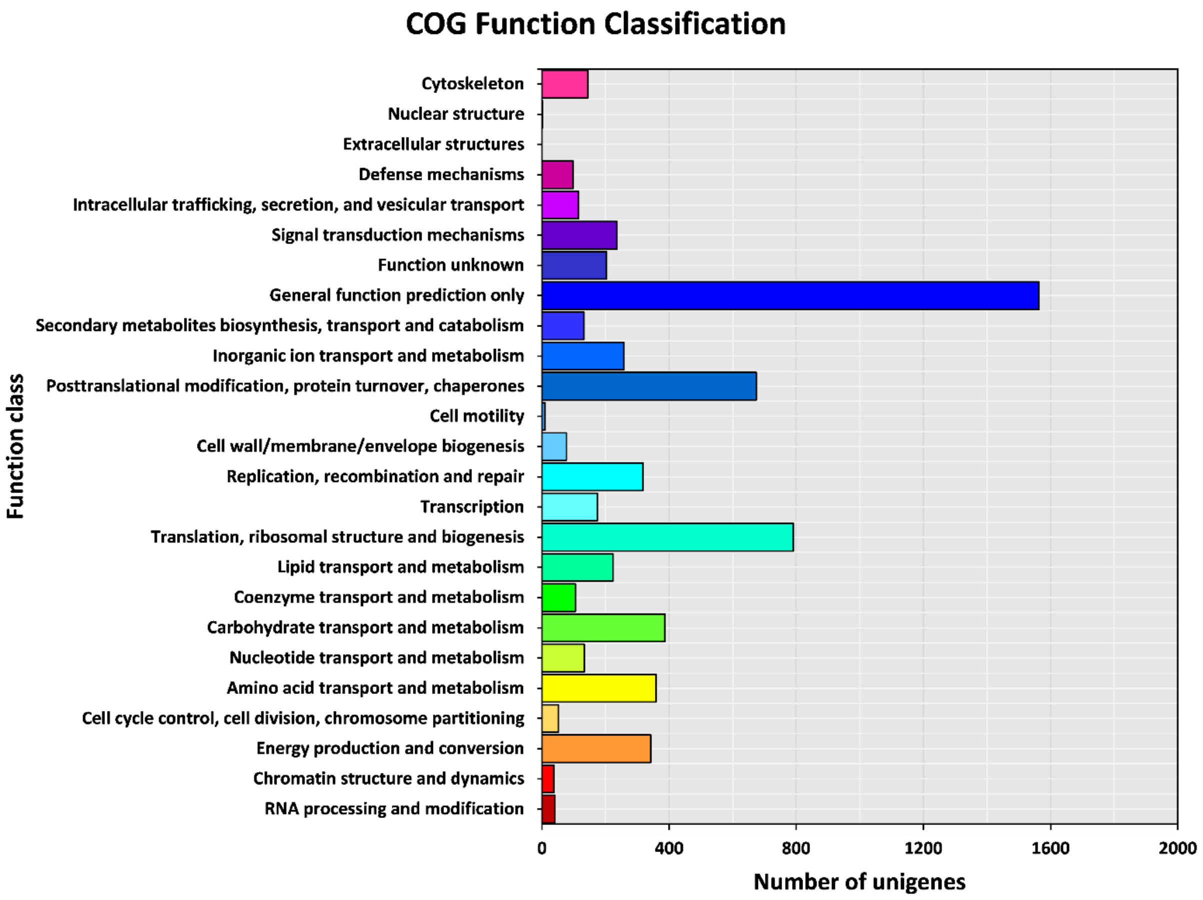

Figure 3 Clusters of orthologous group (COG) function classification for *B. similaris*.

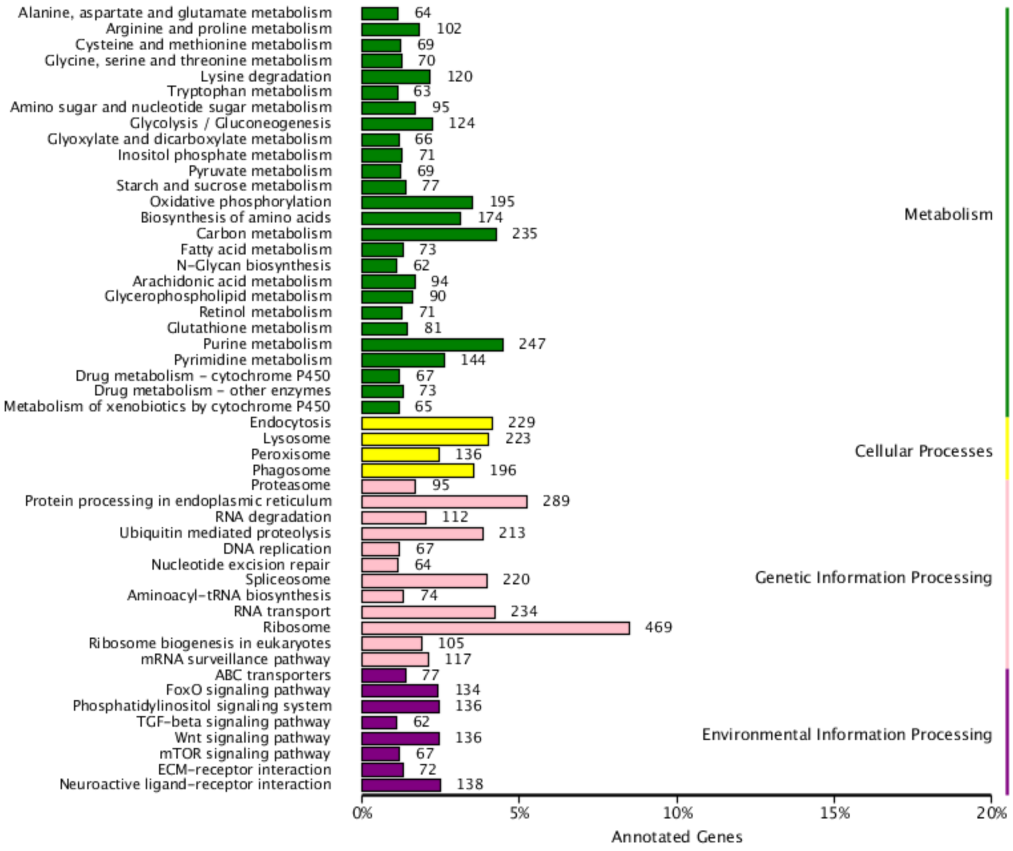

Figure 4 Functional annotation based on KEGG pathway.

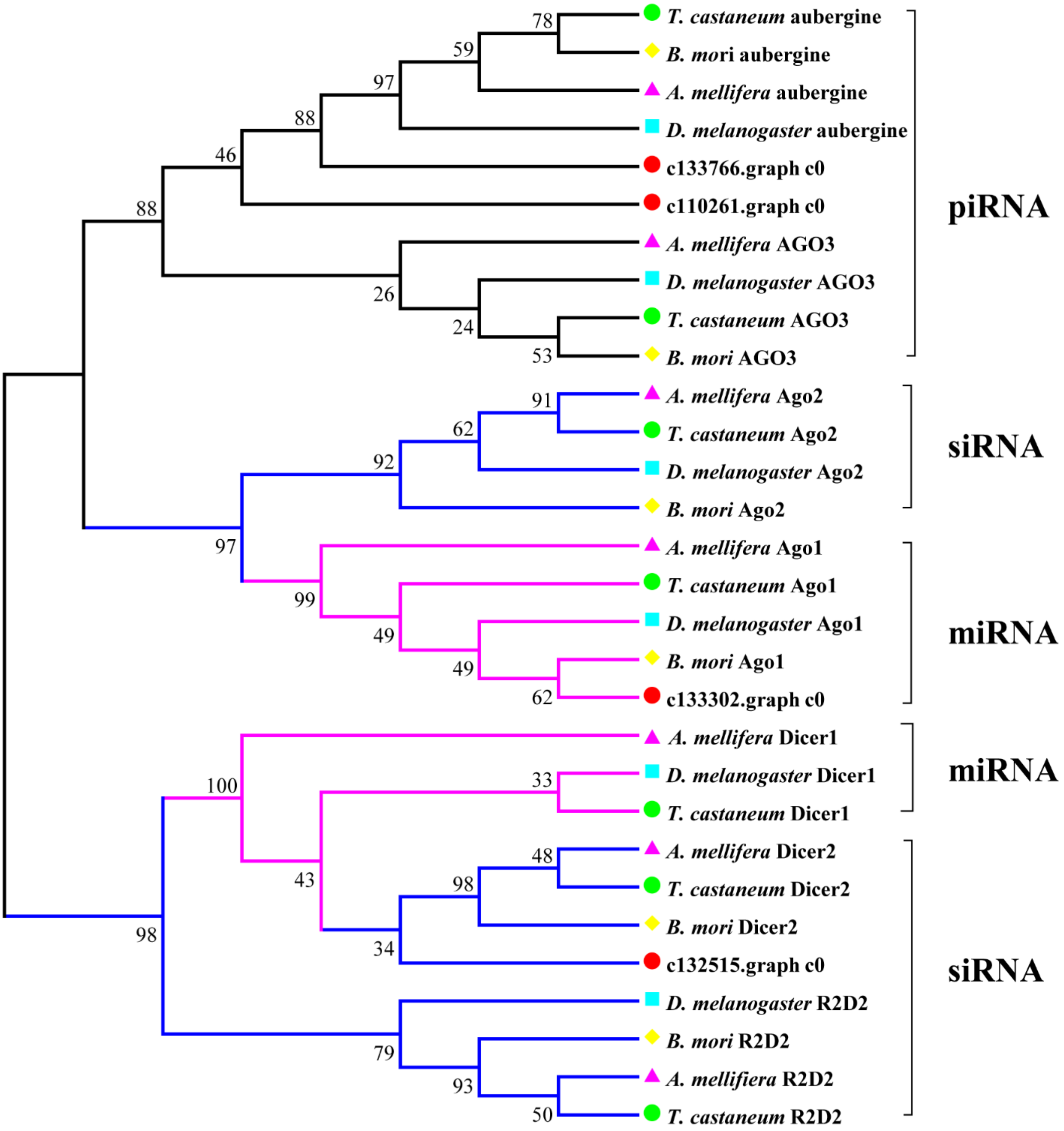

Figure 5 Phylogenetic analysis of putative RNAi pathway core machinery. Neighbor-joining tree of *B. similaris, Apis mellifera*, *Bombyx mor, Tribolium castaneum* and *Drosophila melanogaster*. Numbers at each branch indicate the percentage of the times a node was supported in 1000 bootstrap pseudo-replications by the neighbor-joining method. The sequences used for phylogenetic tree construction were listed in Supplementary Table 7.

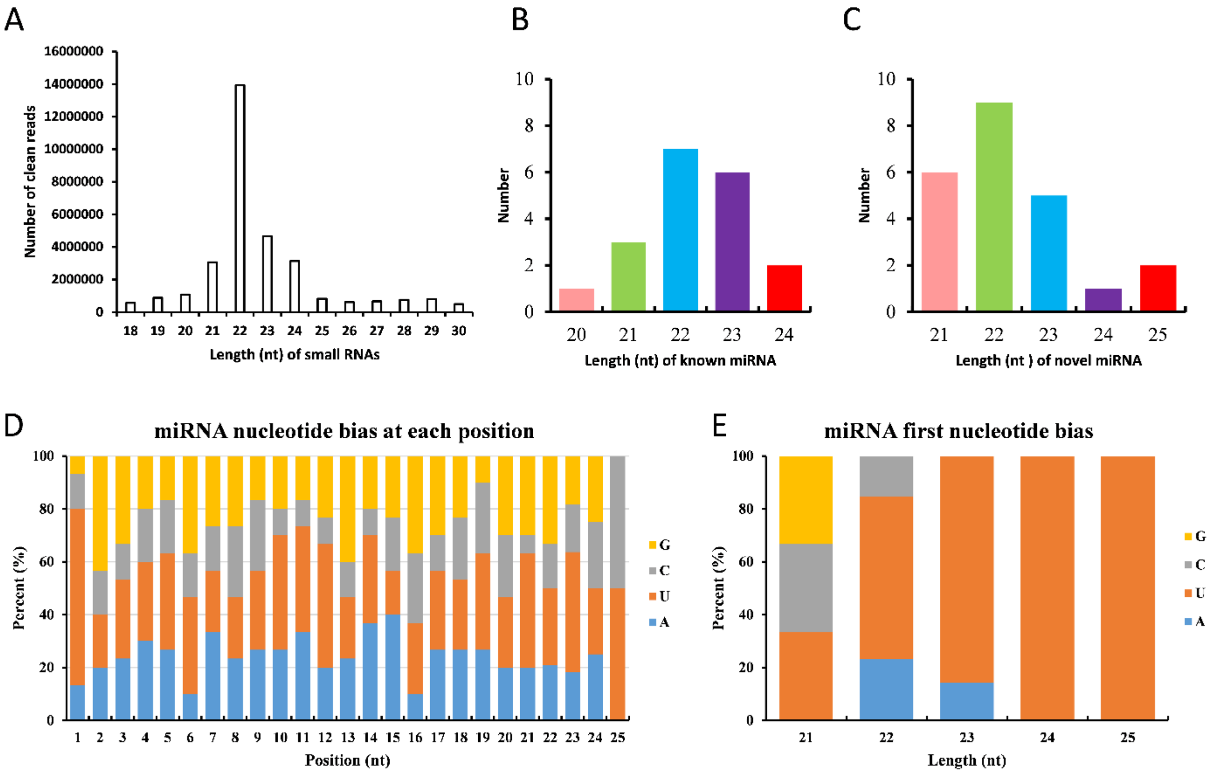

Figure 6 Analysis of miRNAs length and base bias of *B. similaris*. **(A)** Length of all miRNAs. **(B)** Length of known miRNAs. **(C)** Length of novel miRNAs. **(D)** Analysis of the nucleotide bias at each position of the miRNAs. **(E)** Analysis of the nucleotide bias at the first position of the miRNAs.
